# Serum-Amyloid-A to High-Density-Lipoprotein-Cholesterol Ratio: superior biomarker for early diagnosis of coronary artery disease with clinically relevant stenoses and development of machine learning diagnostic model

**DOI:** 10.3389/fcvm.2026.1719851

**Published:** 2026-01-30

**Authors:** Zongze Wang, Xuechen Wang, Yuang Cai, Aimin Zhang, Yanli Wang, Lijuan Hu, Yu Guo, Jun Ma

**Affiliations:** 1 Department of Medical Laboratory, Tianjin Hospital of ITCWM Nankai Hospital, Tianjin, China; 2 School of Medicine, Nankai University, Tianjin, China

**Keywords:** coronary artery disease, C-reactive protein, extreme gradient boosting, machine learning, serum amyloid A, Serum-Amyloid-A to High-Density-Lipoprotein-Cholesterol Ratio

## Abstract

**Background:**

This study aimed to evaluate the early diagnostic value of Serum-Amyloid-A to High-Density-Lipoprotein-Cholesterol Ratio (SHR) for coronary artery disease (CAD) with clinically relevant stenoses and develop a machine learning diagnostic model based on eXtreme Gradient Boosting (XGBoost).

**Methods and results:**

Data from 1,108 CAD patients (with coronary luminal diameter stenosis ≥50% or evidence of functional myocardial ischemia) and 962 controls were retrospectively analyzed. Receiver operating characteristic (ROC) analysis showed SHR (area under the curve (AUC) = 0.769) outperformed C-reactive protein (CRP) (*p* = 0.006) and Serum amyloid A (SAA) (*p* < 0.001). Four XGBoost models were constructed, and the best model (CRP + SAA + SHR + 13 other variables) achieved an AUC of 0.876. SHR correlated nonlinearly with age (*p* < 0.001), and its diagnostic efficacy was higher in younger patients (40 years old, OR = 16.29) than in older adults (80 years old, OR = 4.37). Machine learning models can address the decline in diagnostic capability of SHR in the elderly population.

**Conclusion:**

SHR is a superior composite biomarker for early diagnosis of CAD with clinically relevant stenoses, outperforming CRP and SAA. Machine learning model integrating multiple indicators shows excellent diagnostic performance. Elevated SHR indicates higher CAD risk in younger individuals, providing a new strategy for early screening of CAD with clinically relevant stenoses.

## 1 Introduction

Coronary artery disease (CAD) continues to be a major contributor to global morbidity and mortality (1). Its prevalence shows significant geographical variability, with higher age-standardized incidence rates observed in low- and middle-income countries than in high-income nations which reflects disparities in healthcare accessibility and risk factor management (2). In the United States alone, CAD affects 7.2% of adults aged ≥20 years, with an estimated annual economic burden > US200 billion (3). Cardiovascular disease remains the leading cause of mortality in China currently, where CAD constitutes the fourth most common cause of death (4). Gender disparities persist, with men demonstrating higher age-adjusted incidence rates compared to premenopausal women, though this gap narrows post-menopause (5).

Early diagnosis of CAD is crucial. Even with advancements in optical coherence tomography (OCT) and intravascular ultrasound (IVUS), challenges remain. OCT and IVUS, though gold standards for cap thickness measurement, are invasive, cost-prohibitive, and unsuitable for asymptomatic populations, limiting their utility to high-risk cohorts (6). Coronary computed tomography angiography (CCTA), while valuable for risk stratification, struggles to differentiate lipid-rich necrotic cores from mixed calcified lesions due to limited contrast resolution, leading to underestimation of plaque vulnerability (7).

Atherosclerosis, which is confirmed by multiple studies, is intricately linked to systemic micro-inflammation, which drives plaque initiation, progression, and destabilization (8). Central to this chronic inflammatory process are inflammatory biomarkers such as C-reactive protein (CRP) and serum amyloid A (SAA).

CRP is an acute-phase reactant produced by hepatocytes in response to interleukin-6 (IL-6) (9). C-reactive protein inhibits the production of nitric oxide in endothelial cells, increases the expression of endothelial cell adhesion molecules, and promotes the aggregation of monocytes in atherosclerotic plaques, thereby leading to plaque instability (10). High-sensitivity CRP (hs-CRP) assays are now routinely used to stratify cardiovascular risk, because of its precise quantification of CRP concentrations within the normal to mildly elevated range (11, 12).

SAA is another hepatocyte-derived inflammatory mediator. Many of its functions are related to atherosclerosis and affect the promotion of chemotaxis, the migration of blood cells to lesions, the reduction of high-density lipoprotein (HDL)'s anti-inflammatory ability, and the retention of lipoproteins in the vascular wall (13). The development of “high sensitivity chemiluminescence assays” and “automated immunoturbidimetric platforms” has enabled precise quantification of SAA at lower thresholds, significantly improving its diagnostic utility (14, 15), which has revolutionized the management of infectious diseases and autoinflammatory diseases (16–19).

Recent studies have increasingly focused on computationally derived composite biomarkers such as the monocyte to high-density lipoprotein cholesterol ratio (MHR) and neutrophil to high-density lipoprotein cholesterol ratio (NHR) (20, 21). These integrated indices offer significant advantages over single biomarkers by combining key parameters from distinct physiological pathways, while demonstrating cost-effectiveness and high reproducibility in clinical practice (22).

Inspired by this, we propose a novel composite biomarker: Serum-Amyloid-A to High-Density-Lipoprotein-Cholesterol Ratio (SHR). This ratio dynamically links an acute-phase protein (SAA) with a key lipid metabolism component (High-density lipoprotein cholesterol (HDL-C)), offering a new perspective for the early detection of CAD by integrating inflammation activation with lipid dysregulation.

Concurrently, machine learning (ML)-based diagnostic prediction models have emerged as a major research focus. Among these, the eXtreme Gradient Boosting (XGBoost) algorithm stands out due to its advantages including robust handling of high-dimensional data, built-in regularization to prevent overfitting, automatic handling of missing values, and high computational efficiency through parallel processing (23).

This study aims to systematically compare the individual diagnostic efficacy of CRP, SAA, SHR and other laboratory test indicators for CAD (with clinically relevant stenoses, rather than CAD in general) and further evaluate their performance within XGBoost prediction model; subsequently, we delve into the potential clinical value of SHR for the early diagnosis of this specific CAD subtype. Our findings confirm the superiority of SHR, providing valuable theoretical groundwork for the early non-invasive diagnosis of high-risk CAD that requires clinical intervention.

## 2 Patients and methods

### 2.1 Study subjects

The data of test results of patients admitted to the cardiology department of Tianjin Nankai Hospital from January 2024 to April 2025 were analyzed retrospectively. The patient information was concealed for the purpose of patient privacy protection.

First, it is clarified that the CAD defined in this study specifically refers to the subtype with clinically relevant coronary artery stenosis (luminal diameter stenosis ≥50%), which is consistent with the criteria specified in the Chinese Guidelines for the Diagnosis and Management of Patients with Chronic Coronary Syndrome (CCS) 2024.

**Figure 1:**
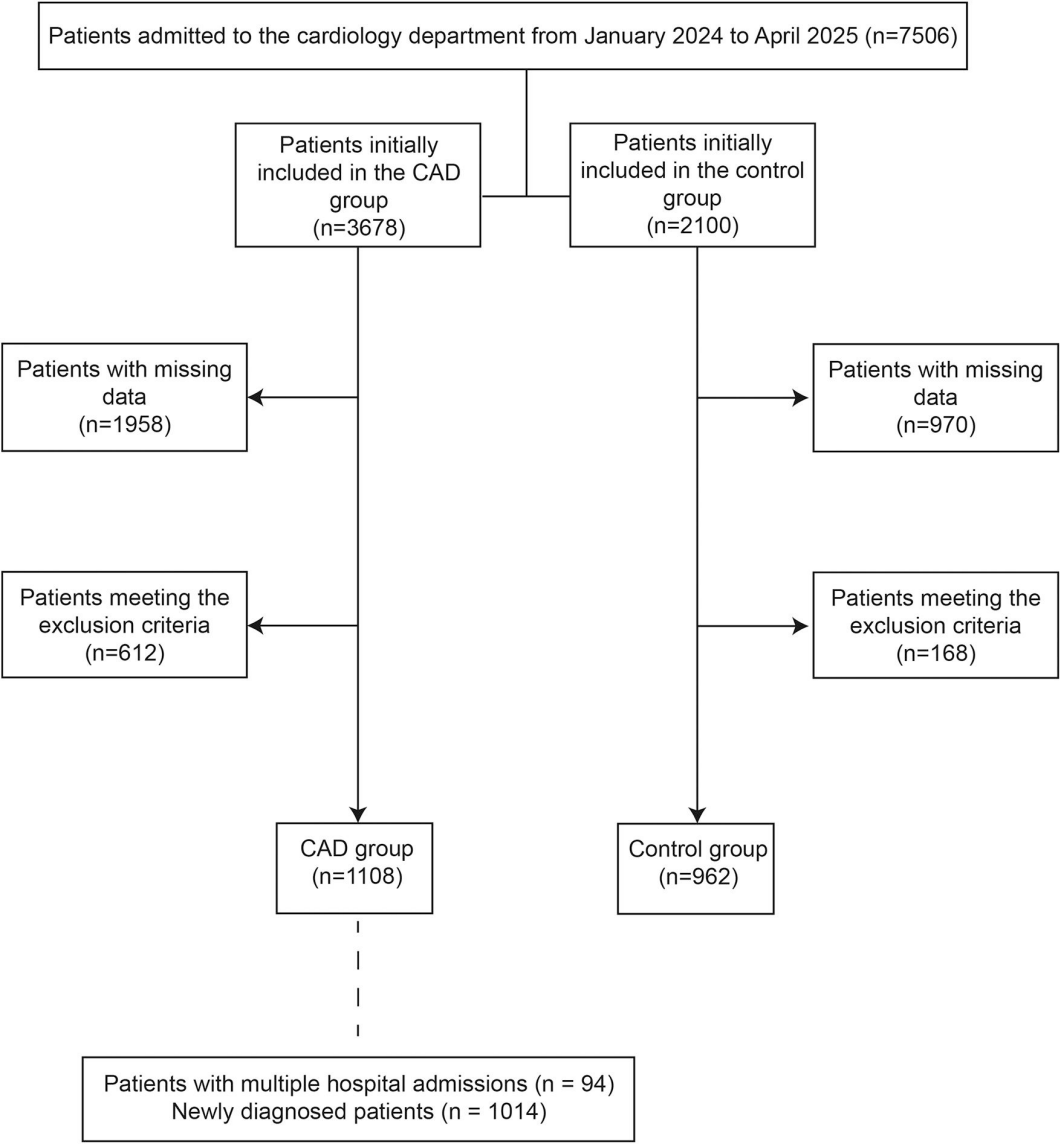
The flow chart of study population selection. Patients with multiple hospital admissions: patients who have been admitted to Tianjin Nankai Hospital multiple times and have documented standard treatment records. Newly diagnosed patients: Patients newly diagnosed with coronary heart disease at Tianjin Nankai Hospital who have no prior treatment records at Tianjin Nankai Hospital.

According to the above guidelines, the inclusion criteria for the CAD group are as follows: (1) Age >18 years; (2) Presence of coronary artery anatomical stenosis (coronary angiography or coronary computed tomography angiography showing luminal diameter stenosis ≥50%) or evidence of functional myocardial ischemia (positive myocardial ischemia indicated by stress electrocardiogram, stress myocardial perfusion imaging, stress echocardiography, or other relevant examinations); (3) Typical or atypical angina pectoris symptoms (e.g., retrosternal/precordial oppressive or distending pain, which may radiate to the left shoulder/neck/mandible, induced by exertion or emotional excitement and relieved by rest; or atypical symptoms such as chest tightness, shortness of breath, fatigue, etc.), with persistent or recurrent symptoms (disease duration ≥2 weeks and stable condition without acute exacerbation); (4) Asymptomatic but with definite high-risk factors for CAD (e.g., hypertension, diabetes mellitus, dyslipidemia, smoking, family history of premature CAD, etc.), accompanied by evidence of coronary artery anatomical stenosis or functional myocardial ischemia. The inclusion criteria for the control group are as follows: (1) Age >18 years; (2) Seeking medical advice due to chest tightness or chest pain pending investigation, or for suspected cardiovascular abnormalities found during a physical examination (such as abnormal blood lipids or blood pressure fluctuations); (3) Less than 50% coronary artery stenosis confirmed by coronary computed tomography angiography (CCTA) or invasive coronary angiography; (4) No objective evidence of myocardial ischemia (negative exercise ECG, no reversible defects on myocardial perfusion imaging). Exclusion criteria are as follows: (1) Serious comorbidities such as advanced cancer, severe liver and kidney dysfunction, thyroid dysfunction, end-stage heart failure, severe infections, autoimmune diseases, etc.; (2) History of percutaneous coronary intervention or coronary artery bypass surgery; (3) Drug or alcohol dependence; (4) Pregnancy or lactation; (5) Serious mental illness. Ultimately, 2,070 patients were included, among whom 1,108 cases in CAD group and 962 cases control group (Figure 1).

### 2.2 Patient data

The following data were obtained by consulting the medical records: (1) Sex and age; (2) Laboratory test results including CRP, SAA, White blood cell count (WBC), Neutrophil% (NEUT%), Lymphocyte% (LYMPH%), Monocyte% (MONO%), Neutrophil count (NEUT), Lymphocyte count (LYMPH), Monocytes count (MONO), Platelet count (PLT), Aspartate transaminase (AST), Lactate dehydrogenase (LDH), α-hydroxybutyratedehydrogenase (HBDH), Creatine kinase myocardial band (CK-MB), Cholesterol (CHOL), HDL-C, Low-density lipoprotein cholesterol (LDL-C); (3) Composite indicators including SHR, NHR, Lymphocyte to high-density lipoprotein cholesterol ratio (LHR), MHR, Systemic immune-inflammation index (SII), Neutrophil to lymphocyte ratio (NLR), Platelet to lymphocyte ratio (PLR), Lymphocyte to monocyte ratio (LMR), Neutrophil to platelet ratio (NPR), Systemic inflammatory response index (SIRI).

These variables were selected due to their precise quantifiability, and the data can be directly extracted without the risk of privacy disclosure, ensuring the accuracy and reliability of the input data for the machine learning model.

All composite indicators in this article were calculated as follows:

$$\mathrm{NHR}=\frac{\mathrm{NE}}{\mathrm{HDL}\text{-}C}$$

$$\mathrm{LHR}=\frac{\mathrm{LYMPH}}{\mathrm{HDL}\text{-}C}$$

$$\mathrm{MHR}=\frac{\mathrm{MONO}}{\mathrm{HDL}\text{-}C}$$

$$\mathrm{SII}=\frac{\mathrm{PLT}\times \;\mathrm{NE}}{\mathrm{LYMPH}}$$

$$\mathrm{NLR}=\frac{\mathrm{NE}}{\mathrm{LYMPH}}$$

$$\mathrm{PLR}=\frac{\mathrm{PLT}}{\mathrm{LYMPH}}$$

$$\mathrm{LMR}=\frac{\mathrm{LYMPH}}{\mathrm{MONO}}$$

$$\mathrm{NPR}=\frac{\mathrm{NE}}{\mathrm{PLT}}$$

$$\mathrm{SIRI}=\frac{\mathrm{NE}\times \mathrm{MONO}}{\mathrm{LYMPH}}$$

To eliminate the scale differences between SAA and HDL-C, SHR was calculated as follows:

$$\mathrm{SHR}=\ln\frac{\mathrm{SAA}+1}{\mathrm{HDL}\text{-}C}$$

The study was approved by the Institutional Ethics Committee of Tianjin Nankai Hospital on March 24, 2025 (NKYY_YXKT_IRB_2025_028_01)

### 2.3 Sample measurements

**Figure 2:**
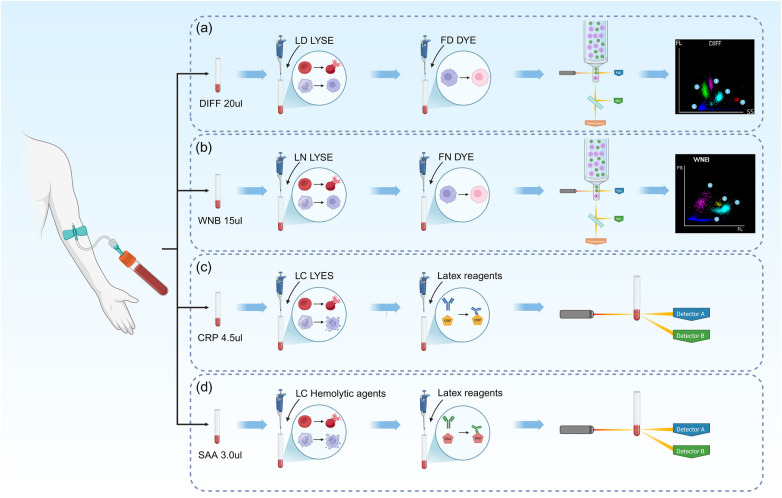
The process of sample measurements. **(a)** the process of DIFF channel; **(b)** the process of WNB channel; **(c)** the process of hs-CRP measurement; **(d)** the process of SAA measurement. Created in BioRender, licensed under Academic License.

Blood routine tests, hs-CRP test and SAA test were carried out using BC-7500 Auto Hematology Analyzers (Mindray, Shenzhen, China). The process is shown in Figure 2.

Blood HDL-C levels and other biochemical test were carried out by AU5800 Series Chemistry Analyzers (Beckman Coulter, Suzhou, China).

### 2.4 Experiment design

This study consists of two sequential analyses. The first experiment has two parts, and the second experiment has three parts.

The first analysis compares the diagnostic capabilities of SHR and other laboratory test indicators individually and within predictive models. In the first part of this analysis, receiver operating characteristic (ROC) curves were plotted, and the area under the curve (AUC) with 95% confidence intervals (CI) was calculated. Optimal cut-off values were determined using the Youden index. Differences in AUCs were assessed using DeLong's test.

The second part of this analysis develops an XGBoost-based diagnostic model. After correlation analysis (Spearman's rank) and feature selection via Least Absolute Shrinkage and Selection Operator (LASSO) regression, the dataset was split into training (70%) and validation (30%) sets. Four models were built: selected features plus CRP, SAA, SHR, or all three. Hyperparameters were tuned using grid search cross-validation (CV) and manual fine-tuning. Models were evaluated by ROC analysis, calibration curves, Hosmer-Lemeshow test, and decision curve analysis (DCA). The best model was interpreted using Shapley Additive exPlanations (SHAP) values.

The second analysis explores how SHR's diagnostic ability relates to age. The first part uses a Generalized Additive Model (GAM) and an Ordinary Linear Regression (OLR) model to examine the SHR-age relationship. The second part stratifies patients by age quartiles and uses logistic regression to calculate adjusted odds ratios (OR) for each group. GAM-based visualizations include marginal effect plots, partial dependence plots (PDP), heatmaps, and individual conditional expectation (ICE) curves. The third part analyzes SHAP values for SHR across age groups using box plots and trend curves. Two heatmaps are plotted: one for case distribution by age and SHAP value, and another for SHAP values by age and SHR.

All analyses were performed by R software (version 4.5.1). Continuous variables that follow a normal distribution are expressed as mean ± SD. Continuous variables that do not follow a normal distribution are expressed as median [first quartile (Q1), third quartile (Q3)]. Categorical variables are expressed as counts (percentages). Missing data were handled by listwise deletion. All tests were 2-sided, and *p* *<* 0.05 was considered statistically significant.

## 3 Results

### 3.1 Baseline characteristics comparison between patients with multiple hospital admissions and newly diagnosed patients in CAD group

There were 94 patients with multiple hospital admissions and 1,014 newly diagnosed patients in CAD group. As shown in Table 1, apart from patients with multiple hospital admissions had lower levels of CHOL and LDL-C, there were no other differences between the two subgroups. Since there were no differences in key indicators such as CRP, SAA, HDL-C and SHR between the groups, subsequent studies will consider the CAD group as a whole.

**Table 1:** Baseline characteristics comparison between patients with multiple hospital admissions and newly diagnosed patients in CAD group.

Variables	Total (*n* = 1,108)	Patients with multiple hospital admissions (*n* = 94)	Newly diagnosed patients (*n* = 1,014)	*p*
Sex, *n* (%)				0.206
female	487 (44)	35 (37)	452 (45)	
male	621 (56)	59 (63)	562 (55)	
Age, Median (Q1,Q3)	70 (62, 76)	71.5 (61.25, 76.75)	70 (62, 76)	0.637
CRP, Median (Q1,Q3)	3.29 (1.36, 8.24)	3.04 (1.2, 6.26)	3.3 (1.39, 8.46)	0.258
SAA, Median (Q1,Q3)	3.98 (2.18, 11.17)	4.02 (2.41, 7.84)	3.97 (2.18, 11.48)	0.636
SHR, Median (Q1,Q3)	1.53 (1.19, 2.52)	1.59 (1.26, 2.15)	1.53 (1.19, 2.56)	0.965
WBC, Median (Q1,Q3)	6.47 (5.35, 7.88)	6.46 (5.32, 7.68)	6.49 (5.35, 7.88)	0.42
NEUT%, Mean ± SD	67.46 ± 10.27	67.28 ± 10.76	67.48 ± 10.22	0.86
LYMPH%, Mean ± SD	23.09 ± 9.03	23.11 ± 9.31	23.09 ± 9.01	0.986
MONO%, Median (Q1,Q3)	6.6 (5.4, 7.9)	6.7 (5.6, 7.95)	6.6 (5.4, 7.9)	0.494
PLT, Median (Q1,Q3)	208 (169, 250)	202.5 (167.25, 247.5)	209 (169, 250)	0.803
AST, Median (Q1,Q3)	21 (17, 27)	19.5 (16, 24)	21 (17, 27)	0.032
LDH, Median (Q1,Q3)	181 (156, 215.25)	182 (156, 208.25)	181 (156, 216)	0.857
HBDH, Median (Q1,Q3)	116 (101, 137)	115 (103.25, 132.75)	116 (101, 137.75)	0.644
CK, Median (Q1,Q3)	72 (48, 111.25)	66 (43.25, 97.75)	72 (49, 112.75)	0.08
CKMB, Median (Q1,Q3)	1.8 (1.34, 2.43)	1.66 (1.25, 2.21)	1.82 (1.35, 2.45)	0.091
CHOL, Median (Q1,Q3)	3.96 (3.22, 4.76)	3.58 (2.9, 4.28)	3.99 (3.25, 4.79)	<0.001***
HDL-C, Median (Q1,Q3)	1.06 (0.9, 1.24)	1.04 (0.87, 1.19)	1.06 (0.9, 1.24)	0.437
LDL-C, Median (Q1,Q3)	2.62 (2.01, 3.26)	2.23 (1.8, 2.78)	2.67 (2.03, 3.3)	<0.001***
NEUT, Median (Q1,Q3)	4.37 (3.41, 5.46)	4.31 (3.24, 5.24)	4.37 (3.42, 5.48)	0.602
LYMPH, Median (Q1,Q3)	1.43 (1.04, 1.88)	1.38 (1, 1.85)	1.44 (1.04, 1.89)	0.547
MONO, Median (Q1,Q3)	0.42 (0.33, 0.54)	0.43 (0.32, 0.54)	0.42 (0.33, 0.54)	0.773
NHR, Median (Q1,Q3)	4.12 (3.01, 5.54)	4.18 (3.12, 5.54)	4.11 (3.01, 5.53)	0.912
LHR, Median (Q1,Q3)	1.34 (0.94, 1.86)	1.35 (0.89, 2.03)	1.33 (0.94, 1.84)	0.997
MHR, Median (Q1,Q3)	0.41 (0.29, 0.55)	0.4 (0.31, 0.51)	0.41 (0.29, 0.55)	0.762
SII, Median (Q1,Q3)	618.96 (421.33, 942.65)	646.86 (423.09, 1,027.48)	614.75 (421.18, 934.32)	0.557
NLR, Median (Q1,Q3)	2.92 (2.09, 4.47)	2.89 (1.95, 4.69)	2.92 (2.1, 4.45)	0.992
PLR, Median (Q1,Q3)	144.47 (110.9, 196.21)	153.12 (114.84, 202.02)	143.81 (110.12, 195.66)	0.268
LMR, Median (Q1,Q3)	3.38 (2.48, 4.64)	3.43 (2.42, 4.65)	3.38 (2.48, 4.64)	0.968
NPR, Median (Q1,Q3)	0.02 (0.02, 0.03)	0.02 (0.02, 0.03)	0.02 (0.02, 0.03)	0.224
SIRI, Median (Q1,Q3)	1.26 (0.81, 2.01)	1.26 (0.81, 2.09)	1.26 (0.81, 2)	0.917

*** *p* < 0.001.

### 3.2 Baseline characteristics of all subjects

There were 1,108 cases in the CAD group and 962 cases in the control group. The baseline characteristics of all the subjects in this study are shown in Table 2. CAD group had higher percentage in male, higher age, CRP, SAA, and SHR. The differences in WBC, NEUT%, LYMPH%, MONO%, PLT, AST, LDH, HBDH, CK-MB, CHOL, HDL-C, LDL-C, NE, LYMPH, MONO, NHR, LHR, MHR, SII, NLR, PLR, LMR, NPR, SIRI were also observed between CAD group and control group.

**Table 2:** Baseline characteristics of all subjects.

Variables	Total (*n* = 2,070)	Control group (*n* = 962)	CAD group (*n* = 1,108)	*p*
Sex, *n* (%)				<0.001***
Female	1,019 (49)	532 (55)	487 (44)	
Male	1,051 (51)	430 (45)	621 (56)	
Age, Median (Q1,Q3)	67 (59, 74)	64 (53.25, 71)	70 (62, 76)	<0.001***
CRP, Median (Q1,Q3)	1.95 (0.84, 4.48)	1.20 (0.60, 2.25)	3.29 (1.36, 8.24)	<0.001***
SAA, Median (Q1,Q3)	2.78 (2.00, 5.28)	2.00 (2.00, 3.11)	3.98 (2.18, 11.17)	<0.001***
SHR, Median (Q1,Q3)	1.28 (1.01, 1.73)	1.10 (0.90, 1.33)	1.53 (1.19, 2.52)	<0.001***
WBC, Median (Q1,Q3)	6.33 (5.15, 7.65)	6.13 (4.99, 7.39)	6.47 (5.35, 7.88)	<0.001***
NEUT%, Mean ± SD	65.36 ± 10.29	62.94 ± 9.77	67.46 ± 10.27	<0.001***
LYMPH%, Median (Q1,Q3)	25.3 (19.3, 32.0)	28.2 (22.4, 34.4)	23.25 (16.6, 28.9)	<0.001***
MONO%, Median (Q1,Q3)	6.3 (5.2, 7.5)	6.0 (5.0, 7.1)	6.6 (5.4, 7.9)	<0.001***
PLT, Median (Q1,Q3)	210 (175, 250)	212.5 (181, 250)	208 (169, 250)	0.020*
AST, Median (Q1,Q3)	20 (17, 26)	20 (16, 25)	21 (17, 27)	0.028*
LDH, Median (Q1,Q3)	174 (152, 204)	169 (149, 193)	181 (156, 215.25)	<0.001***
HBDH, Median (Q1,Q3)	113 (99, 131)	108 (97, 124)	116 (101, 137)	<0.001***
CK, Median (Q1,Q3)	72 (50, 106)	72 (52, 101.75)	72 (48, 111.25)	0.881
CKMB, Median (Q1,Q3)	1.71 (1.29, 2.22)	1.60 (1.25, 2.07)	1.80 (1.34, 2.43)	<0.001***
CHOL, Median (Q1,Q3)	4.27 (3.47, 5.11)	4.55 (3.81, 5.41)	3.96 (3.22, 4.76)	<0.001***
HDL-C, Median (Q1,Q3)	1.10 (0.94, 1.03)	1.16 (0.99, 1.37)	1.06 (0.90, 1.24)	<0.001***
LDL-C, Median (Q1,Q3)	2.82 (2.18, 3.48)	3.04 (2.43, 3.71)	2.62 (2.01, 3.26)	<0.001***
NEUT, Median (Q1,Q3)	4.13 (3.15, 5.19)	3.83 (2.94, 4.86)	4.37 (3.41, 5.46)	<0.001***
LYMPH, Median (Q1,Q3)	1.54 (1.15, 2.01)	1.68 (1.31, 2.12)	1.43 (1.04, 1.88)	<0.001***
MONO, Median (Q1,Q3)	0.39 (0.30, 0.50)	0.36 (0.29, 0.45)	0.42 (0.33, 0.54)	<0.001***
NHR, Median (Q1,Q3)	3.72 (2.64, 5.09)	3.27 (2.40, 4.55)	4.12 (3.01, 5.54)	<0.001***
LHR, Median (Q1,Q3)	1.39 (1.00, 1.90)	1.43 (1.06, 1.94)	1.34 (0.94, 1.86)	<0.001***
MHR, Median (Q1,Q3)	0.36 (0.25, 0.50)	0.31 (0.22, 0.41)	0.41 (0.29, 0.55)	<0.001***
SII, Median (Q1,Q3)	547.74 (372.05, 810.03)	481.42 (325.17, 687.01)	618.96 (421.33, 942.65)	<0.001***
NLR, Median (Q1,Q3)	2.59 (1.84, 3.72)	2.23 (1.61, 3.11)	2.92 (2.09, 4.47)	<0.001***
PLR, Median (Q1,Q3)	134.82 (104.03, 179.82)	124.85 (99.32, 163.46)	144.47 (110.9, 196.21)	<0.001***
LMR, Median (Q1,Q3)	3.99 (2.89, 5.33)	4.63 (3.60, 6.02)	3.38 (2.48, 4.64)	<0.001***
NPR, Median (Q1,Q3)	0.02 (0.01, 0.03)	0.02 (0.01, 0.02)	0.02 (0.02, 0.03)	<0.001***
SIRI, Median (Q1,Q3)	1.03 (0.64, 1.61)	0.81 (0.54, 1.23)	1.26 (0.81, 2.01)	<0.001***

* *p* < 0.05,

*** *p* < 0.001.

### 3.3 Comparative analysis of SHR and other variables

**Figure 3:**
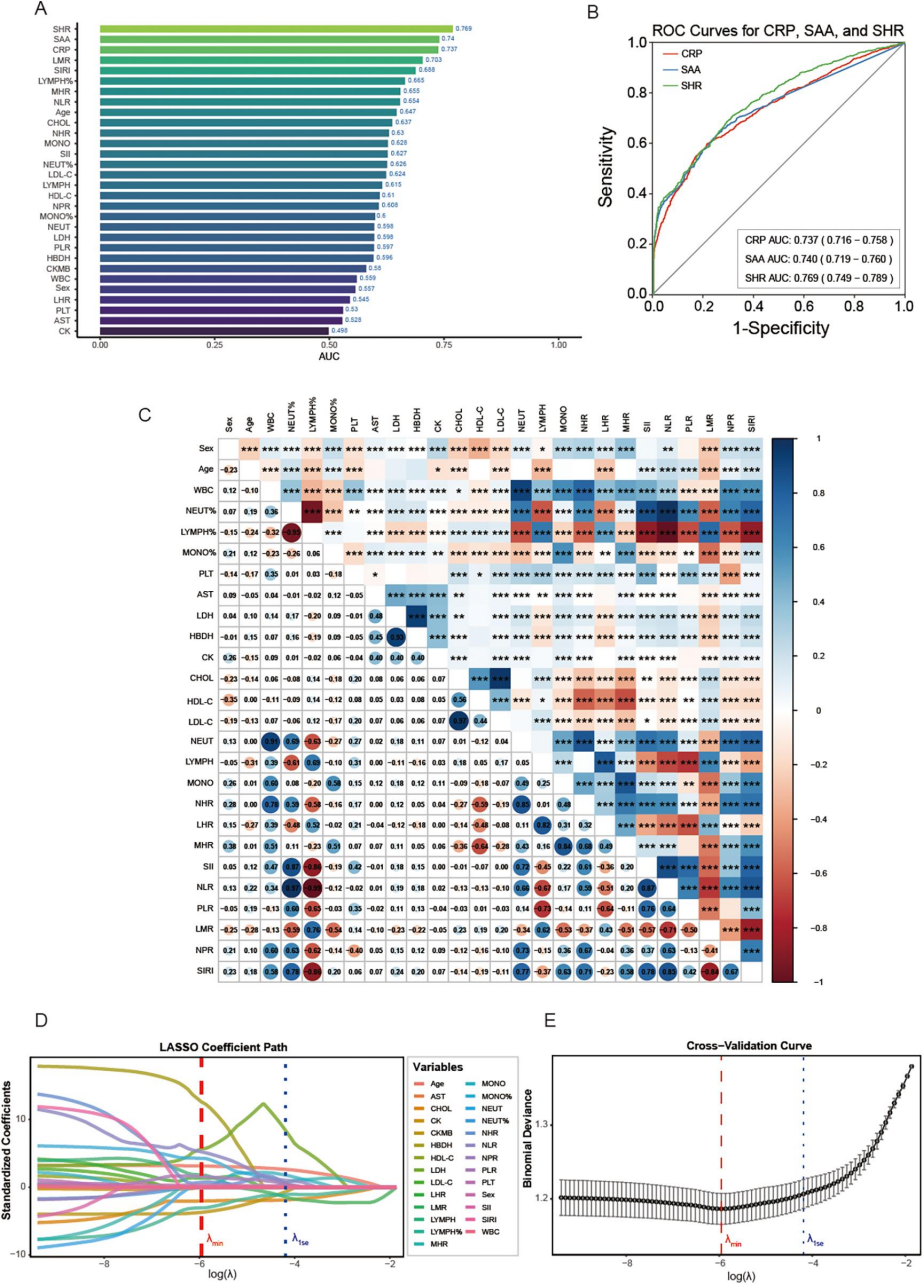
**(A)**: Bar chart of the AUC for all included indicators; **(B)**: ROC curves for CRP, SAA, and SHR; **(C)**: correlation analysis of 27 candidate variables (the bluer the color, the stronger the positive correlation; the redder the color, the stronger the negative correlation. * indicates that there is a statistically significant relation between the two variables. * *p* *<* 0.05, ** *p* *<* 0.01, *** *p* *<* 0.001); **(D)**: LASSO coefficient path; **(E)**: Cross-validation curve (The red dashed line represents the log(*λ*) value corresponding to the minimum cross-validation error; The blue dotted line represents the log(*λ*) value corresponding to the minimum cross-validation error +1 standard error).

As shown in Figure 3A, the AUCs of CRP, SAA, and SHR are the top three, with SHR showing the optimal performance. As shown in Figure 3B and Table 3, CRP achieved an AUC of 0.737 (95% CI: 0.716–0.758) with 59.7% sensitivity and 78.8% specificity at the optimal cut-off of 2.52 mg/L. SAA achieved an AUC of 0.740 (95% CI: 0.719–0.760), exhibiting 65.9% sensitivity and 72.5% specificity at 2.96 mg/L. SHR exhibited the highest diagnostic efficacy with an AUC of 0.769 (95% CI: 0.749–0.789), reaching 68.5% sensitivity and 71.0% specificity at the threshold of 1.28. DeLong's test revealed that the AUC of SHR was significantly higher than both CRP (*p* *=* 0.006) and SAA (*p* *<* 0.001), while no significant statistical difference was observed between CRP and SAA (*p* *=* 0.831).

**Table 3:** ROC curves for CRP, SAA, and SHR.

Variables	AUC (95%CI)	Sensitivity	Specificity	Cut-off
CRP	0.737 (0.716–0.758)	0.597	0.788	2.52
SAA	0.740 (0.719–0.760)	0.659	0.725	2.96
SHR	0.769 (0.749–0.789)	0.685	0.710	1.28

### 3.4 Correlation analysis

As shown in Figure 3C, spearman's rank correlation analysis of 27 candidate variables revealed significant statistical associations among multiple variables (*p* *<* 0.05), but the absolute value of the correlation coefficients was low for the vast majority (|r| < 0.5), indicating that the correlation strength is generally weak. Notably, LYMPH% exhibits a strong negative correlation with NEUT% (r = −0.95, *p* *<* 0.001), HBDH exhibits a strong positive correlation with LDH (r = 0.93, *p* *<* 0.001), CHOL exhibits a moderate positive correlation with HDL-C (r = 0.56, *p* *<* 0.001) and a strong positive correlation with LDL-C (r = 0.97, *p* *<* 0.001); In addition, the correlation between composite indicators is also strong (|r| > 0.5), for they are calculated in a similar way. The correlation between the remaining indicators is significant, but the clinical significance may be limited.

### 3.5 Baseline characteristics comparison between the training set and the validation set

There were 1,449 cases in the training set and 621 cases in the validation set. The baseline characteristics of training set and validation set are shown in Table 4. There was no statistical difference between the training set and the validation set for all indicators and diagnosis.

**Table 4:** Baseline characteristics comparison between the training set and the validation set.

Variables	Total (*n* = 2,070)	Validation set (*n* = 621)	Training set (*n* = 1,449)	*p*
Sex, *n* (%)				0.173
female	1,019 (49)	291 (47)	728 (50)	
Male	1,051 (51)	330 (53)	721 (50)	
Age, Median (Q1,Q3)	67 (59, 74)	68 (59, 75)	67 (59, 73)	0.281
CRP, Median (Q1,Q3)	1.95 (0.84, 4.48)	1.76 (0.75, 4.26)	2 (0.85, 4.59)	0.147
SAA, Median (Q1,Q3)	2.78 (2, 5.28)	2.68 (2, 5.06)	2.84 (2, 5.32)	0.343
SHR, Median (Q1,Q3)	1.28 (1.01, 1.73)	1.28 (1.02, 1.72)	1.28 (1.01, 1.75)	0.989
WBC, Median (Q1,Q3)	6.33 (5.15, 7.65)	6.41 (5.12, 7.69)	6.29 (5.15, 7.63)	0.688
NEUT%, Mean ± SD	65.36 ± 10.29	65.52 ± 10.26	65.29 ± 10.3	0.638
LYMPH%, Median (Q1,Q3)	25.3 (19.3, 32)	24.6 (18.8, 32.4)	25.4 (19.5, 31.8)	0.457
MONO%, Median (Q1,Q3)	6.3 (5.2, 7.5)	6.3 (5.2, 7.5)	6.3 (5.2, 7.5)	0.731
PLT, Median (Q1,Q3)	210 (175, 250)	207 (174, 256)	211 (175, 249)	0.993
AST, Median (Q1,Q3)	20 (17, 26)	20 (17, 28)	20 (17, 25)	0.206
LDH, Median (Q1,Q3)	174 (152, 204)	177 (154, 203)	174 (151, 204)	0.369
HBDH, Median (Q1,Q3)	113 (99, 131)	113 (99, 129)	113 (98, 132)	0.998
CK, Median (Q1,Q3)	72 (50, 106)	74 (50, 112)	71 (50, 105)	0.262
CKMB, Median (Q1,Q3)	1.71 (1.29, 2.22)	1.7 (1.29, 2.27)	1.71 (1.27, 2.22)	0.820
CHOL, Median (Q1,Q3)	4.27 (3.47, 5.11)	4.19 (3.35, 5.06)	4.28 (3.49, 5.12)	0.119
HDL-C, Median (Q1,Q3)	1.1 (0.94, 1.3)	1.08 (0.92, 1.3)	1.11 (0.95, 1.3)	0.104
LDL-C, Median (Q1,Q3)	2.82 (2.18, 3.48)	2.75 (2.14, 3.45)	2.83 (2.21, 3.51)	0.106
NEUT, Median (Q1,Q3)	4.13 (3.15, 5.19)	4.17 (3.1, 5.29)	4.11 (3.17, 5.16)	0.631
LYMPH, Median (Q1,Q3)	1.54 (1.15, 2.01)	1.58 (1.13, 1.99)	1.53 (1.16, 2.02)	0.758
MONO, Median (Q1,Q3)	0.39 (0.3, 0.5)	0.39 (0.31, 0.51)	0.39 (0.3, 0.5)	0.517
NHR, Median (Q1,Q3)	3.72 (2.64, 5.09)	3.88 (2.58, 5.18)	3.67 (2.66, 5.04)	0.356
LHR, Median (Q1,Q3)	1.39 (1, 1.9)	1.4 (0.99, 1.89)	1.38 (1, 1.91)	0.840
MHR, Median (Q1,Q3)	0.36 (0.25, 0.5)	0.36 (0.25, 0.53)	0.35 (0.25, 0.49)	0.189
SII, Median (Q1,Q3)	547.74 (372.05, 810.03)	570.69 (374.38, 828.9)	540.66 (371.18, 803.28)	0.372
NLR, Median (Q1,Q3)	2.59 (1.84, 3.72)	2.65 (1.81, 3.85)	2.57 (1.85, 3.63)	0.474
PLR, Median (Q1,Q3)	134.82 (104.03, 179.82)	136.92 (104.26, 183.29)	134.22 (103.94, 178.71)	0.415
LMR, Median (Q1,Q3)	3.99 (2.89, 5.33)	3.9 (2.85, 5.33)	4.05 (2.91, 5.33)	0.394
NPR, Median (Q1,Q3)	0.02 (0.01, 0.03)	0.02 (0.01, 0.03)	0.02 (0.01, 0.03)	0.714
SIRI, Median (Q1,Q3)	1.03 (0.64, 1.61)	1.06 (0.66, 1.66)	1.02 (0.64, 1.59)	0.389
Diagnosis, *n* (%)				0.122
Control group	962 (46)	272 (44)	690 (48)	
CAD group	1,108 (54)	349 (56)	759 (52)	

### 3.6 Result of LASSO regression

Employing the “minimum cross-validation error +1 standard error” (1se) criterion, LASSO regression with 10-fold CV achieved optimal trade-off between model complexity and generalizability. Figure 3D illustrates coefficient shrinkage paths where 27 variables progressively zeroed out with increasing log(*λ*). Figure 3E displays the CV error curve, peaking at log(*λ*) = −5.96, while the conservative 1se criterion corresponded to log(*λ*) = −4.20. At this threshold, 14 variables were excluded, retaining 13 variables. Sex, age, LYMPH%, MONO%, LDH, CKMB, CHOL, MONO, NHR, MHR, NLR, PLR, and LMR demonstrated strong weights, whereas WBC and 13 other variables showed marginal contributions. This selection substantially mitigated multicollinearity effects, yielding a robust feature subset for subsequent modeling.

### 3.7 Comparative analysis of four XGBoost prediction models

**Figure 4:**
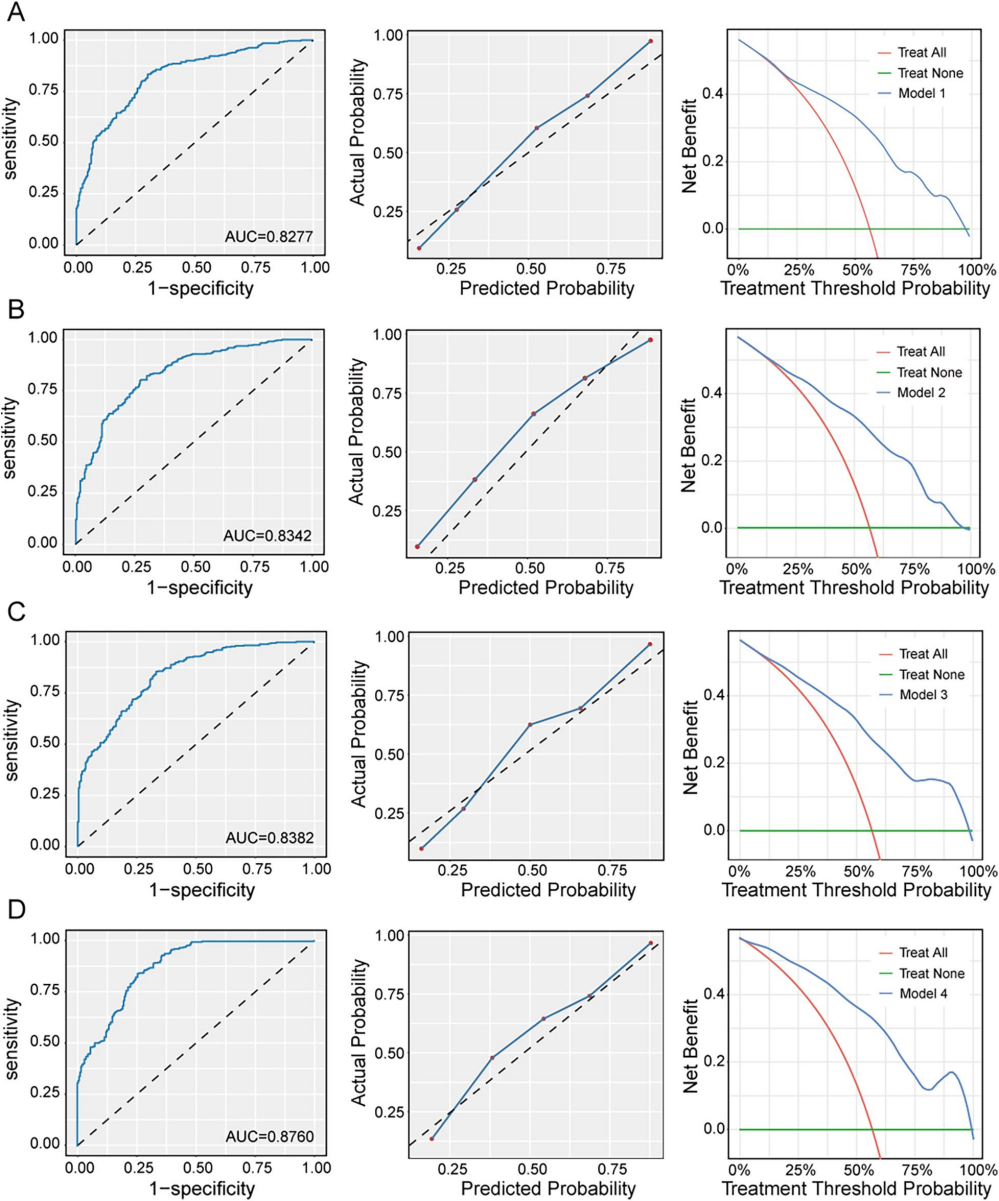
ROC, calibration curve, and decision curve of 4 XGBoost prediction models; **(A)**: model 1 (CRP + 13 other variables); **(B)**: model 2 (SAA + 13 other variables); **(C)**: model 3 (SHR + 13 other variables); **(D)**: model 4 (CRP + SAA + SHR + 13 other variables).

Four XGBoost models demonstrated outstanding diagnostic performance in the independent validation set. As depicted in Figures 4A–D and Table 5: ROC analysis of Model 4 revealed superior discriminative ability with an AUC of 0.876 (95% CI: 0.849–0.903), significantly higher than Model 1 (AUC = 0.828; Delong test, *p* *<* 0.001).The AUC of Model 2 (AUC = 0.834; Delong test, *p* *=* 0.789) or Model 3 (AUC = 0.838; Delong test, *p* *=* 0.482) was not significantly higher than Model 1. The calibration curve of Model 3 (slope = 1.390, intercept = 0.164) and Model 4 (slope = 1.245, intercept = 0.137) exhibited a slope close to the ideal diagonal, and Hosmer-Lemeshow test for Model 3 (*p* *=* 0.077) and Model 4 (*p* *=* 0.081) confirmed excellent agreement between predicted probabilities and actual risks. Decision curve analysis validated clinical utility: Model 4 provided higher net benefit than “treat-all” and “treat-none” strategies across the 1%–96% threshold probability range, which was wider than Model 1 (1%–93%), Model 2 (1%–90%), and Model 3 (1%–94%). Collectively, these results indicate Model 4 had the highest accuracy and clinical applicability.

**Table 5:** ROC, calibration curve, and decision curve of 4 XGBoost prediction models; model 1: CRP + 13 other variables; model 2: SAA + 13 other variables; model 3: SHR + 13 other variables; model 4: CRP + SAA + SHR + 13 other variables.

Models	ROC		Calibration curve			Decision curve
	AUC (95% CI)	Delong test	Slope	intercept	Hosmer-Lemeshow test	Threshold probability range
Model 1	0.828 (0.796–0.860)	Reference	1.349	0.166	*p* *=* 0.009**	0.01–0.93
Model 2	0.834 (0.803–0.866)	*p* *=* 0.798	0.689	0.072	*p* < 0.001***	0.01–0.90
Model 3	0.838 (0.808–0.869)	*p* *=* 0.482	1.390	0.164	*p* *=* 0.077	0.01–0.94
Model 4	0.876 (0.849–0.903)	*p* *<* 0.001***	1.245	0.137	*p* *=* 0.081	0.01–0.96

** *p* < 0.01,

*** *p* < 0.001.

### 3.8 Interpretability analysis using SHAP for the best prediction model

**Figure 5:**
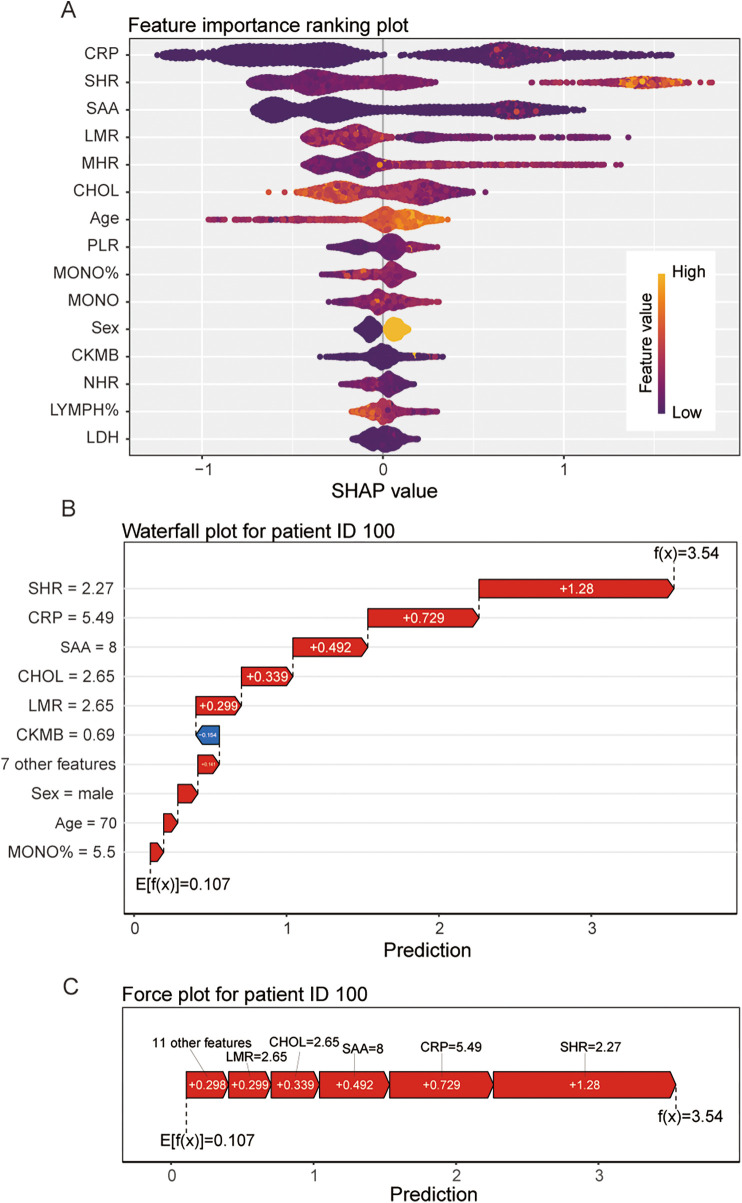
Interpretability analysis for model 4; **(A)**: feature importance ranking plot; **(B)**: waterfall plot for patient ID 100; **(C)**: force plot for patient ID 100.

Systematic interpretation via SHAP elucidated the decision logic of Model 4. As shown in Figure 5A, feature importance ranking identified CRP, SHR, SAA, LMR, and MHR as primary drivers, with contributions substantially exceeding CHOL and other indicators.

For a high-risk patient (ID: 100, predicted probability 97.2%), the waterfall plot (Figure 5B) deconstructed individualized decision pathways: SHR elevation (SHAP = 1.21) dominated risk, synergistically amplified by CRP (SHAP = 0.746) and SAA (SHAP = 0.472). The force plot (Figure 5C) visualized how baseline risk (0.271) escalated to 3.53 through cumulative feature effects. This interpretability framework provides actionable biological insights for clinical intervention.

### 3.9 Relationship between SHR and age

**Figure 6:**
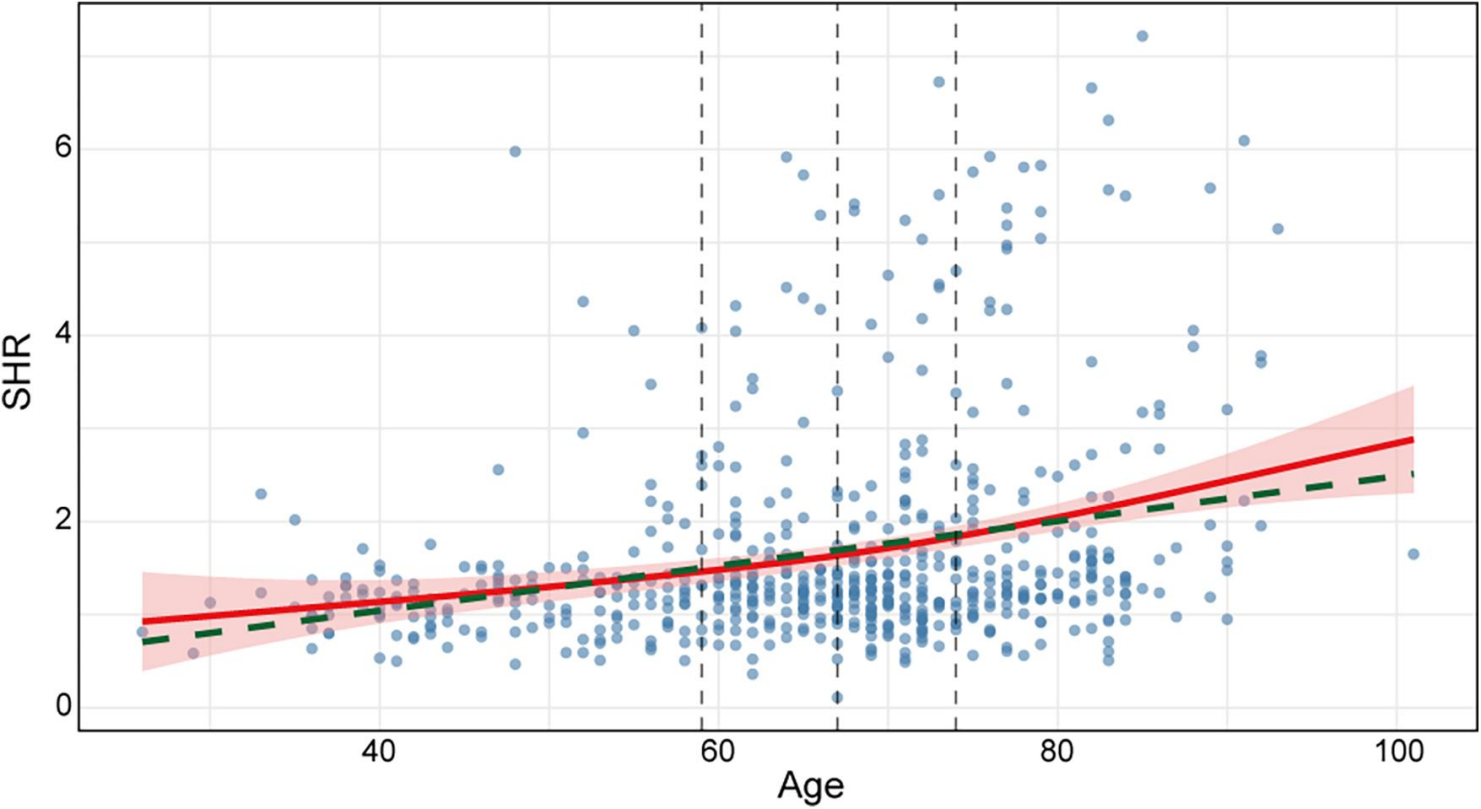
Scatter plot for the relationship between SHR and age; Red solid line indicates GAM-fitted curve (shaded area indicating 95% confidence interval); green dashed line indicates OR-fitted curve for reference; black dashed line indicates quartile of age (59, 67, and 74).

The results demonstrated a significant positive correlation between SHR and age (Spearman's rank correlation, *p* *<* 0.001). GAM analysis further revealed a nonlinear ascending trend of SHR with advancing age (nonlinear term *p* *<* 0.001), exhibiting accelerated increase beyond 70 years. Model fitness tests indicated superior performance of GAM (R² = 0.076, AIC = 1912, EDF = 2.14, *p* *<* 0.001) over Ordinary Linear Regression (R² = 0.072, AIC = 1,913). Visualization through scatter plots distinctly illustrated this relationship (Figure 6): the GAM-fitted curve showed progressive elevation of SHR with age, whereas the linear regression trend systematically underestimated SHR increments in both younger and older adults.

### 3.10 Relationship between the diagnostic capability of SHR and age

**Figure 7:**
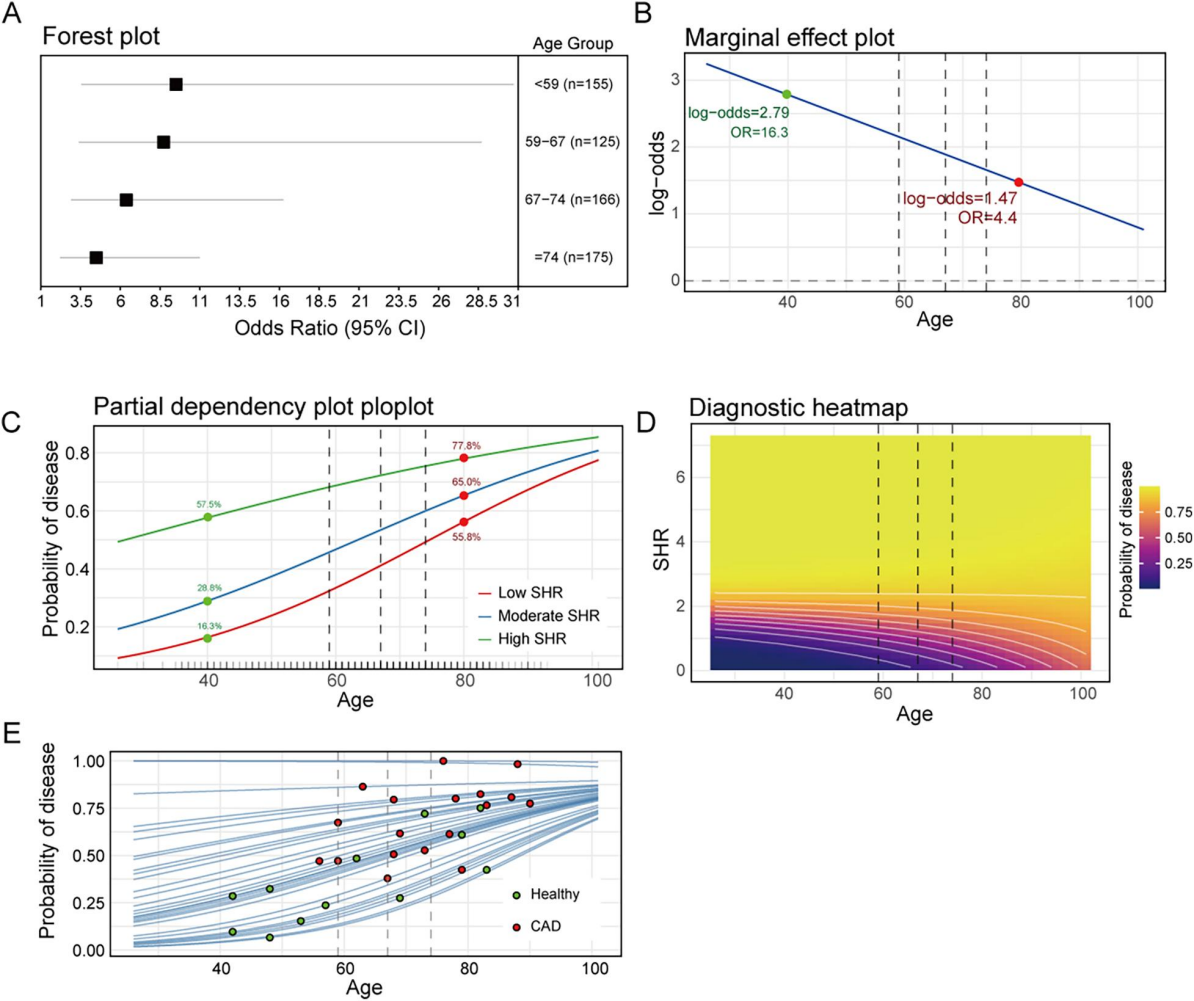
Relationship between the diagnostic capability of SHR and age; **(A)**: forest plot of age-quartile groups; **(B)**: marginal effect plot, the green dot indicates the log-odds value corresponding to the age of 40, and the red dot indicates the log-odds value corresponding to the age of 80; **(C)**: partial dependency plot, the green dot indicates the probability of disease corresponding to the age of 40, the red dot indicates the probability of disease corresponding to the age of 80; **(D)**: diagnostic heatmap; **(E)**: individual conditional expectation curve; black dashed line indicates quartile of age (59, 67, and 74).

The diagnostic capability of SHR for CAD demonstrated significant age-dependent attenuation. As shown in Figure 7A, forest plot of age-quartile groups revealed progressively declining odds ratios (Q1: OR = 9.50 (3.58–30.68); Q2: OR = 8.72 (3.42–28.67); Q3: OR = 6.38 (2.94–16.22), Q4: OR = 4.49 (2.25–10.97)). GAM confirmed this relationship (tensor product smooth term ti(SHR, Age): *p* *=* 0.002). The marginal effect plot (Figure 7B) shows that the predictive efficacy of SHR exhibits a declining trend. At the age of 40, log-odds = 2.790 (OR = 16.29), indicating that for every 1 unit increase in SHR, the risk of disease increases by 1,529%. At the age of 80, log-odds = 1.474 (OR = 4.37), where the risk only increases by 337%. This instead suggests the significant value of SHR in the younger population. The partial dependency plot (Figure 7C) shows that when SHR rises from low (first quartiles) to moderate (median) and high levels (third quartiles) at age 40, the prevalence probability increases by 12.5% and 44.5%, respectively. At the age of 80, when SHR increased from low level to moderate level and high level, the probability of disease increased by 9.2% and 22.0%, respectively. Diagnostic heatmap (Figure 7D) and Individual Conditional Expectation curve (Figure 7E) also visually demonstrated this downward trend of the diagnostic capability of SHR for CAD.

### 3.11 Age-dependent contribution of SHR within XGBoost model

**Figure 8:**
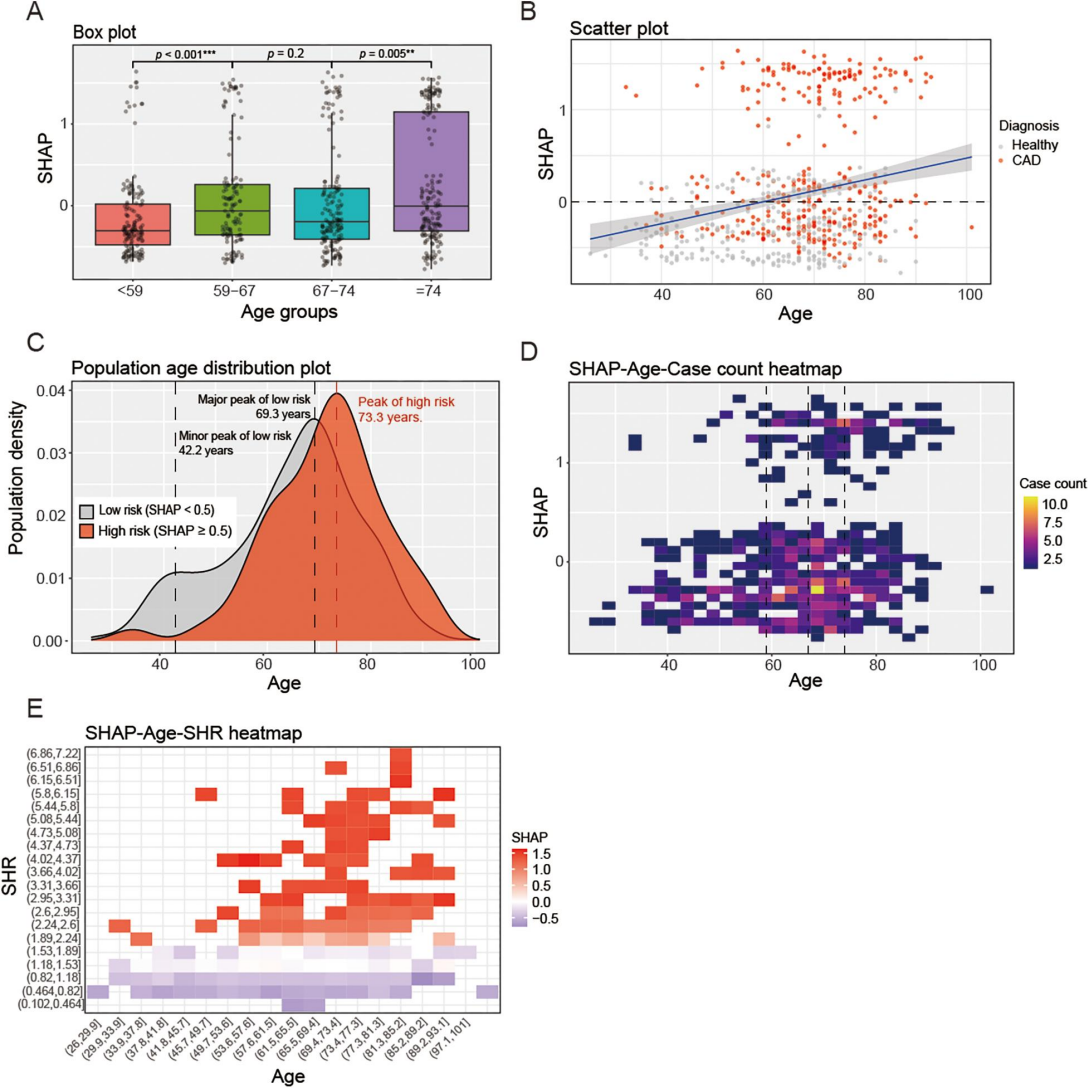
Age-dependent contribution of SHR within XGBoost model; **(A)**: Box plot of age-quartile groups, * *p* *<* 0.05, ** *p* < 0.01, *** *p* < 0.001; **(B)**: scatter plot, blue solid line indicates GAM-fitted curve (shaded area indicating 95% confidence interval); **(C)**: population age distribution plot, black dashed line indicates the minor peak and major peak of low risk group, red dashed line indicates the peak of high risk group; **(D)**: SHAP-Age-case count heatmap, black dashed line indicates quartile of age (59, 67, and 74); **(E)**: SHAP-Age-SHR heatmap.

XGBoost interpretability analysis revealed a significant positive correlation between the diagnostic contribution of SHR (SHAP values) and age (GAM smoothing term: *p* *<* 0.001). Box plot (Figure 8A) across age quartiles demonstrated ascending SHAP values. The median of group 2 was significantly higher than that of group 1 (*p* *<* 0.001), group 3 had no significant change compared with group 2 (*p* *=* 0.2), and the median of group 4 was significantly higher than that of group 3 (*p* *=* 0.005). Scatter plot (Figure 8B) overlaid with GAM curves confirmed this trend. Population age distribution plot (Figure 8C) revealed fundamental divergence between low-risk (SHAP* <* 0.5, gray) and high-risk (SHAP ≥ 0.5, red) density curves (Mann–Whitney, *p* *<* 0.001). The low-risk group exhibited bimodal distribution—minor peak at 42.2 years, major peak at 69.2 years—whereas the high-risk group showed delayed peak at 73.3 years. This aligns with SHAP-age-case count heatmap (Figure 8D): cases with elevated SHAP (>0.5) located around 73 years, and cases with low SHAP values dispersed across a wider age range. SHAP-age-SHR heatmap (Figure 8E) identified a “high-age-high-SHR” zone with SHAP values >0.5, directly driving superior CAD detection accuracy in the elderly.

## 4 Discussion

The clinical application of SAA as a routine biomarker was limited in previous decades due to insufficient sensitivity and specificity. These limitations were addressed by latex-enhanced immunoturbidimetric assays (LEIAs), and both CRP and SAA can be detected together on the same instrument, thereby increasing throughput and reducing labor and reagent costs. Furthermore, improved detection accuracy allows for the identification of chronic microinflammation, which provides a new idea for the early diagnosis of CAD.

The pathogenesis of atherosclerosis is intrinsically linked to chronic inflammation, involving a complex interplay between acute-phase proteins, innate immune cells, and lipoprotein modifications, which collectively drive endothelial dysfunction, plaque formation, and disease progression.

SAA is an acute-phase reactant like CRP, which enhances monocytes and leukocyte recruitment, adhesion and activation by inducing IL-1, IL-6, and MCP-1 production (13). Both CRP and SAA synergistically amplify the pro-inflammatory milieu by stimulating the secretion of cytokine, further perpetuating hepatic acute-phase protein synthesis.

HDL usually exerts a protective effect on atherosclerosis through reverse cholesterol transport and anti-inflammatory signaling, and low HDL-C levels are considered a cardiovascular risk factor. Even worse, under inflammatory conditions, SAA replaces apolipoprotein A-I (apoA-I) on HDL particles, converting them from anti-inflammatory to pro-inflammatory mediators, and causing low HDL-C level (24, 25). As a result, SAA-enriched HDL loses its protective effect, further accelerate the progression of atherosclerosis (26). Therefore, the level of SHR can not only integrate the levels of inflammation and lipid metabolism but also reflect the interaction between SAA and HDL. Our study is consistent with the research by Zewinger S et al., Soria-Florido MT et al., and McEneny J et al., all indicating that an increase in SAA and a decrease in HDL-C elevate the risk of cardiovascular disease (27–29). Research by Stock EO et al. also employed machine learning methods, combining HDL-C with inflammatory biomarkers, and demonstrated a stronger capability for predicting CAD risk (30).

The effects of commonly used medications on SHR are also relatively limited. Although statin therapy can significantly reduce the levels of LDL-C and non-HDL-C, it has little impact on the HDL-C in the bloodstream (31). This is consistent with the results we observed in 3.1. However, there is still very little research on the relationship between non-steroidal anti-inflammatory drugs and SAA levels. According to the research by Du JL, patients with short-duration type 2 diabetes underwent intensive multifactorial integrated therapy targeting hyperglycemia, hypertension, dyslipidemia and aspirin, showed a reduction in SAA, which was attributed to the reduction of triglyceride (32). In our observation, patients with multiple hospital admissions did not show a decrease in SAA and SHR.

The diagnostic target of this study is specifically CAD with clinically relevant stenoses. This design aligns with the clinical demand for early screening of high-risk populations that require priority intervention (e.g., drug therapy, percutaneous coronary intervention, or coronary artery bypass grafting), which is of great significance for reducing the incidence of acute myocardial infarction and sudden cardiac death. In the first experiment, this study provides comprehensive evaluation of the diagnostic efficacy of SHR and 29 other laboratory test indicators for CAD through systematic ROC analysis and DeLong's test. The key finding reveals SHR exhibits superior performance. Its AUC (0.769) was significantly higher than both conventional CRP (*p* *=* 0.006) and SAA (*p* *<* 0.001), achieving 68.5% sensitivity and 71.0% specificity at the optimal threshold (1.28), suggesting that SHR may overcome limitations of single acute-phase proteins by integrating inflammation information and lipid metabolism information.

Subsequently, we applied machine-learning techniques to establish a diagnostic prediction model based on XGBoost. The XGBoost diagnostic prediction model developed in this study demonstrates multifaceted advantages through systematic optimization and interpretability frameworks. Technically, integrating LASSO feature selection (retaining 13 key variables via 1se criterion) with CV-based hyperparameter tuning ensured high generalizability (validation AUC 0.876) and robustness (calibration slope 1.245). Clinically, decision curve analysis confirmed actionable net clinical benefit across a wide threshold probability range (1%–96%). Crucially, the SHAP interpretability framework uncovered biologically driven decision logic: the top five indicators (CRP, SHR, SAA, LMR, and MHR) include a large number of composite indicators, including acute phase protein, immune cells and lipid metabolism, which are the three major aspects involved in the occurrence and development of CAD, not only validating known pathological mechanisms but also pinpointing personalized intervention targets (e.g., dual inflammation- dyslipidemia modulation for patient ID100). This trinity of “high-accuracy prediction–visualized interpretation–clinical deployability” signifies a pivotal evolution from predictive models to evidence-based decision support systems.

In the second experiment, our study elucidates the multidimensional relationship between SHR and age with critical clinical implications. According to the research by Franceschi C et al. and Baechle JJ et al., chronic inflammation is a common feature of aging, and cytokine dysregulation is considered a potential mechanism (33–35). Therefore, while inflammation can be regarded as a signal of disease in young people, in the elderly population, regardless of whether they have CAD, the levels of inflammation in their bodies are relatively high, making it unreliable to determine the presence of CAD based on inflammatory markers. This also applies to lipid metabolism indicators (36). Our conclusion is consistent with the conclusions of the above studies.

Physiologically, SHR exhibits nonlinear elevation with aging (GAM: *p* < 0.001), indicating compounded dysregulation of chronic inflammation (increased amyloid A) and lipid metabolism (impaired HDL function). Diagnostically, SHR's capability to identify CAD demonstrates significant age-dependent attenuation—young patients (40 years) with high SHR (third quartiles) had 3.5-fold higher CAD risk than low-SHR peers (first quartiles), whereas the elderly (80 years) showed only 1.4-fold increase. This diagnostic decay provides novel insights for early CAD detection: elevated SHR serves as an independent red flag in youth, while geriatric patients require multi-biomarker integration.

Within machine learning frameworks, the XGBoost model overcame this limitation through feature interactions. Despite reduced specificity of standalone SHR in older adults, its synergy with LMR and MHR enabled automatic down-weighting in non-CAD populations and up-weighting in CAD cases, maintaining diagnostic accuracy. This dynamic reweighting mechanism underscores ML's superiority in deciphering complex biomarker networks.

Our study has limitations. (1) Our study was conducted at one institution and involved retrospective data collection. Future studies should conduct multicenter prospective research. (2) Our study did not include traditional cardiovascular risk factors such as hypertension, diabetes, and smoking. Future research should integrate traditional risk factors to develop more comprehensive predictive models. (3) Our study did not include patients with Acute Coronary Syndrome (ACS), so the conclusions only apply to CCS. Future research can focus on ACS. (4) The research data is only sourced from our hospital's electronic medical records, and it is impossible to obtain patients' medical visits and treatment history from other hospitals, leading to unreliable definitions of whether enrolled patients have received treatment. Future studies should consider the impact of patients' treatment conditions on the research results more comprehensively.

## 5 Conclusion

SHR is an outstanding composite biomarker for early diagnosis of CAD with clinically relevant stenoses, outperforming CRP and SAA. Machine learning model 4 (CRP + SAA + SHR + 13 variables) integrating multiple indicators shows excellent diagnostic performance. Elevated SHR indicates higher CAD risk in younger individuals, providing a new strategy for early screening of CAD with clinically relevant stenoses. Machine learning models overcome the limitations of single metrics in diagnosing older adults and are suitable for clinical translation.

## Data Availability

The data are not publicly available due to their containing information that could compromise the privacy of patients. Requests to access these datasets should be directed to haobbll@outlook.com.
